# Antiageing Mechanisms of a Standardized Supercritical CO_**2**_ Preparation of Black Jack (*Bidens pilosa* L.) in Human Fibroblasts and Skin Fragments

**DOI:** 10.1155/2015/280529

**Published:** 2015-03-26

**Authors:** Gustavo Dieamant, Maria Del Carmen V. Pereda, Cecília Nogueira, Samara Eberlin, Gustavo Facchini, Juliana Tibério Checon, Camila Kappke Cesar, Lilian Mussi, Márcio Antonio Polezel, Divino Martins-Oliveira, Luiz Claudio Di Stasi

**Affiliations:** ^1^Chemyunion Quimica Ltda, Avenida Independência 1501, 18087-101 Sorocaba, SP, Brazil; ^2^Laboratory of Phytomedicines, Pharmacology and Biotechnology (PhytoPharmaTech), Department of Pharmacology, Institute of Biosciences, Universidade Estadual Paulista (UNESP), 18618-000 Botucatu, SP, Brazil

## Abstract

The use of topical retinoids to treat skin disorders and ageing can induce local reactions, while oral retinoids are potent teratogens and produce several unwanted effects. This way, efforts to explore complementary care resources should be supported. Based on this, we evaluate the antiageing effects of a supercritical CO_2_ extract from *Bidens pilosa* L. (BPE-CO_2_A) containing a standardized multicomponent mixture of phytol, linolenic, palmitic, linoleic, and oleic acids. BPE-CO_2_A was assessed for its effects on human dermal fibroblasts (TGF-*β*1 and FGF levels using ELISA; collagen, elastin, and glycosaminoglycan by colorimetric assays, and mRNA expression of RXR, RAR, and EGFr by qRT-PCR) and human skin fragments (RAR, RXR, collagen, elastin, and glycosaminoglycan by immunohistochemical analysis). Levels of extracellular matrix elements, TGF-*β*1 and FGF, and EGFr gene expression were significantly increased by BPE-CO_2_A. The modulation of RXR and RAR was positively demonstrated after the treatment with BPE-CO_2_A or phytol, a component of BPE-CO_2_A. The effects produced by BPE-CO_2_A were similar to or better than those produced by retinol and retinoic acid. The ability to stimulate extracellular matrix elements, increase growth factors, and modulate retinoid and rexinoid receptors provides a basis for the development of preparation containing BPE-CO_2_A as an antiageing/skin-repair agent.

## 1. Introduction

Retinoids are classically defined as compounds that exhibit vitamin A-like effects or bind to nuclear retinoid receptors, exerting their pharmacological effects on gene expression by activating retinoic acid receptors (RARs) or retinoid X receptors (RXRs). RARs are ligand-controlled transcription factors that function as heterodimers with RXRs to regulate cell growth, differentiation, survival, and death [1]. Activation of RAR and RXR has been associated with several diseases, and their ligands are widely used to treat skin disorders, mainly aging and photoaging, acne, and psoriasis, and have been used for cancer therapy and chemoprevention [2, 3].

The beneficial effects of retinoids on ageing skin are attributed to increased dermal function, mainly through the increased production of extracellular matrix components, reduced inflammatory response, and antioxidative properties. These effects are related to epidermal growth factor (EGF) and fibroblast growth factor (FGF), both acting as mediators of the retinoid response in the skin [4]. In addition, the beneficial effects of retinoids on ageing and photoageing include improved fine wrinkling, diminished tactile roughness, improved actinic keratosis, and reduced hyperpigmentation, which are histopathologically associated with epidermal hyperplasia, the compaction of the stratum corneum, the thickening of the granular layer, reduced melanocytic hypertrophy, the restoration of cell polarity, increased angiogenesis, increased new collagen formation, and the normalization of the appearance of elastic tissue [5].

In contrast, the use of topical retinoids to treat skin disorders can induce local reactions including erythema, burning, dryness, desquamation, stinging, and photosensitivity reactions to ultraviolet radiation, whereas the use of oral retinoids is limited because these compounds are potent teratogens and produce several unwanted effects such as cheilitis, cutaneous photosensitivity, conjunctivitis, photophobia, and increased susceptibility to staphylococcal infections [4]. This way, efforts to explore alternative and complementary care resources should be supported [6], and the use of herbal preparations and natural products as a source of new active compounds for complementary and alternative therapies could be an important therapeutic strategy to obtain new products with retinoid-like effects and better management of skin diseases and ageing.

Black Jack (*Bidens pilosa *L. (Asteraceae)) is an annual and ruderal plant originating in South America and is also found in tropical and subtropical regions around the world [7]. This plant is a cosmopolitan herb with high distribution in disturbed areas and is widely used in the traditional medicine of different countries to treat internal and topical inflammatory processes, wounds, insect bites, fungal infections, diabetic ulcers, fever, malaria, inflammation, hepatitis, hemorrhoids, and cancer [7–11]. Recent review showed that* B. pilosa* has anti-inflammatory, immunomodulatory, antimalarial, antitumor, antioxidative, antiulcerogenic, antibacterial, hepatoprotective, and antihypertensive activities [7, 8]. Phytochemical studies indicated that approximately 200 different compounds have been identified and isolated in* B. pilosa*, and among them mainly flavonoids, polyacetylenes, terpenoids, phenylpropanoids, and hydrocarbons were reported [7, 8]. It has been demonstrated that nonpolar chemical constituents of* Bidens pilosa* such as docosahexaenoic acid, phytanic acid, phytol, *α*-tocopherol, ricinoleic acid, and esters of caffeic acid have similar retinoid chemical structures, and some of these compounds have been considered to be natural RXR ligands and modulators of the retinoic acid signalling pathway, directly influencing their transcriptional regulatory activity [12–16].

The study of new products from natural sources, mainly plant species, can be performed through several approaches; however, the supercritical carbon dioxide extraction and fractionation (SC-CO_2_) of natural matter is one of the early and most studied applications with immediate advantages over traditional extraction techniques [17]. It is a flexible process because it allows for the continuous modulation of the solvent power/selectivity and elimination of polluting organic solvents, thereby eliminating the expensive postprocessing of extracts traditionally required for solvent elimination. Moreover, it is more economical and simple than other methods; it has diffusivity that reduces mass transfer limitations and a low surface tension, which allows for the penetration and wetting of pores smaller than those accessible with liquid solvents; it allows extraction at low to moderate temperatures, leaving no solvent residues; and it is environmentally acceptable [17, 18].

Based on the pharmacological properties of* B. pilosa*, such as antioxidative, anti-inflammatory and retinoid-like effects, and its phytochemical composition of mainly nonpolar compounds, the aim of this study was to evaluate a SC-CO_2_ nonpolar extract from the aerial parts of* B. pilosa* to develop an herbal preparation with retinoid-like activity and to potentially use this preparation as an antiageing or skin-repair agent.

## 2. Materials and Methods 

### 2.1. Plant Collection, Plant Extraction, and Gas Chromatography Analysis

*B. pilosa* was cultivated using organic agricultural methods as certified by Ecocert Brazil (Santa Rosa de Lima/Santa Catarina/Br) and submitted to taxonomic identification at Herbarium Irina Gemtchujnikov (Department of Botany, Institute of Biosciences, Universidade Estadual Paulista (UNESP), Botucatu, SP), where a voucher specimen was deposited. The aerial parts were collected, dehydrated in hothouse with air circulation and renewal, and triturated in an industrial mill. A supercritical extraction system (Autoclave Engineers) under the following conditions of 300 bar, 40°C, and CO_2_ flux of 5 L/min was used to generate a nonpolar extract named BPE-CO_2_A.

The extraction procedures of BPE-CO_2_A by GC/MS analysis were made according to following conditions: 100 mg of BPE-CO_2_-A extract with 5 mL of NaOH 0.5 M in methanol was heated in a water bath for 10 min at 37°C. After cooling, 5 mL of NH_4_Cl was added in methanol and heated in a water bath for 5 min at 37°C. 1 mL of BPE-CO_2_A was extracted with 5 mL hexane. After cooling, 1 *μ*L of sample was injected in GC equipment.

GC/MS analysis was performed according to the following conditions: the gas chromatography-mass spectroscopy analysis system consisted of a Gas Chromatograph Focus (Thermo Scientific) equipped with an automatic Triplus sampler and coupled to an ISQ 230ST (Thermo Scientific) mass selective detector. The GC was fitted with an OV-5MS fused silica capillary column (30 m × 0.25 mm × 0.24 *μ*m) and helium was used as the carrier gas at 1 mL/min. Injection was made in splitless mode with injection volume of 1.0 mL/min following a split mode (1 : 50). The injector temperature was 260°C and detector temperature was 200°C. Temperature of column was initially 35°C and then increased to 195°C at 25°C/min, to 205°C at 3°C/min, and finally to 230°C at 8°C/min. Xcalibur software, version 2.1 (Thermo Finigan), was used to acquire and process spectrometric data. Identification of compounds was based on the retention time using NIST08 libraries and comparing with mass spectrum from scientific literature.

### 2.2. Cell Culture

Primary human adult keratinocytes (Invitrogen) and normal human dermal fibroblasts (Cambrex/Lonza) were commercially obtained, grown in specific culture media (keratinocyte growth medium, Epilife-Invitrogen; fibroblast basal medium, Cambrex/Lonza), and cultured in a humidified environment at 37°C and 5% CO_2_. After reaching confluence, the cells were trypsinised, and the total number of cells was counted using a Neubauer camera. After counting, the cells were seeded in culture plates of 24 or 96 wells at densities of 2 × 10^5^ cells/well and 1 × 10^4^ cells/well, respectively.

The apolar extract of* Bidens pilosa* (BPE-CO_2_A) obtained using supercritical fluid extraction was added to the cell cultures at concentrations of 0.05, 0.10, and 0.20 mg/mL. The selection of these doses was based on preliminary cytotoxicity tests using the XTT method (data not shown). As a positive control for comparison with retinoids, we used retinol and retinoic acid at a concentration of 10 mM, according to previous data [19, 20]. After 48-hour incubation, the supernatant and cell lysate were collected for the subsequent measurement of the proposed parameters.

### 2.3. Specimen Skin Collection and Sample Preparation

Human eyelids were surgically removed, dipped in 70% ethanol for 15 seconds, and rinsed 2 times with saline solution. The skin fragments were transferred to a petri dish containing culture medium RPMI-1640 supplemented with foetal bovine serum-Lonza, a combination of antibiotics containing gentamicin and amphotericin-B (gentamicin sulfate/amphotericin-B, Lonza), bovine insulin (Lonza), and growth factor (rhFGF b-r-human fibroblast growth factor-B, Lonza) for a maximum of 24 hours before treatment with BPE-CO_2_A.

BPE-CO_2_A was dissolved in culture medium at 0.5% (a commonly used concentration for retinol) and applied to the skin fragment homogeneously over the entire surface. As positive controls for comparison, we used retinol and retinoic acid, 30 *μ*M and 0.5%, respectively. The samples were kept in contact with the cultures for 48 hours in a humidified atmosphere at 37°C and 5% CO_2_.

### 2.4. TGF-*β* and FGF Evaluation

Transforming growth factor beta (TGF-*β*) and fibroblast growth factor (FGF) were measured in fibroblast culture supernatant according to the instructions for the commercially obtained ELISA kits (R&D Systems), and the concentrations of both growth factors were calculated with reference to a standard curve generated by known concentrations.

### 2.5. Collagen, Elastin, and Glycosaminoglycan Evaluation

Collagen, elastin, and glycosaminoglycan were quantified in the supernatant of fibroblast cultures using commercial colorimetric kits (Biocolor). All extracellular matrix elements were quantified using a standard curve.

### 2.6. RXR, RAR, and EGFr Gene Expression

After human fibroblasts were incubated with predetermined concentrations of BPE-CO_2_A for 48 hours, total RNA was extracted using TriReagent Solution (Applied Biosystems) and quantified using a Quant-iT RNA Assay Kit (Invitrogen) and a Quibit Fluorometer (Invitrogen). The tests were conducted in a StepOnePlus sequence detection system (Applied Biosystems).

The gene expression of RAR, RXR, and EGFR was evaluated using a commercially available kit (TaqMan RNA-to-CT 1-Step, Applied Biosystems) and probes (TaqMan Gene Expression Assays: RAR: Hs0023097_m1; RXR: Hs01067635_m1; EGFR: Hs01075087_m1; B2M: Hs00984230_m1, Applied Biosystems). The B2M (beta-2-microglobulin) gene was used as a reference (endogenous control). The RT-PCR conditions were 48°C for 15 min for reverse transcription and 95°C for 10 minutes for the activation of the Ultra-Pure AmpliTaq Gold DNA Polymerase, followed by 40 cycles of 94°C for 15 seconds and 60°C for 1 minute for denaturation and annealing, respectively.

The relative amount of mRNA was calculated with the 2-ΔΔCT method. Gene expression was considered significant when the expression values were greater than 1.5 times compared to the control. For expression inhibition, values less than 0.5-fold were considered relevant.

### 2.7. Immunohistochemical Assessment in Human Skin Fragments

After 48 hours of incubation with concentrations of BPE-CO_2_A and phytol,* ex vivo* skin fragments were fixed in 4% paraformaldehyde (pH 7.4) for 24 hours and cryoprotected in a 30% sucrose solution for 72 hours. Then, serial sections of 10 *μ*m were collected directly on silanised slides with a cryostat (Leica CN1850).

After the sections were collected, they were washed with PBS. Endogenous peroxidase activity and nonspecific sites were blocked as directed by the Histostain-SP Kit (Zymed Laboratories). Then, the primary anti-RAR, anti-RXR, anti-collagen, anti-elastin, and anti-glycosaminoglycan antibodies were diluted in PBS buffer pH 7.4 containing BSA (5%) and incubated overnight.

The incubation with secondary antibodies, the amplification of immunoreactivity using an avidin-biotin complex, the revelation of the staining using a hydrogen peroxide reaction, and the mounting of the slides were performed following the Histostain-SP Kit instructions.

### 2.8. Histological Sections and Haematoxylin-Eosin Staining

Skin samples were fixed in 10% buffered formalin. After fixation, the material was embedded in paraffin, and serial sections approximately 5 *μ*m thick were made and placed on glass slides. The sections were stained with hematoxylin and eosin (HE). The parameters evaluated were visual qualitative epidermal thickness, the number of viable keratinocytes (basal layer, granular layer, and spinous layer), and barrier condition using the integrity of the stratum corneum.

### 2.9. Ethical Aspects

This study was conducted in accordance with Brazilian law, based on the “Normative Resolution 196/96 CNS/MS,” with the consent of the Committee of Ethics in Research of the School of Medical Sciences, State University of Campinas (UNICAMP). For these experiments, we used skin fragments obtained from blepharoplasty performed at the UNICAMP Hospital.

### 2.10. Statistical Analysis

A parametric method of analysis of variance (ANOVA) was used for statistical analysis followed by Tukey's multiple comparison test. In all groups studied, only those with *P* values less than 0.05 were considered statistically significant.

## 3. Results

### 3.1. Chemical Analysis of BPE-CO_2_A by GC/MS

**Figure 1:**
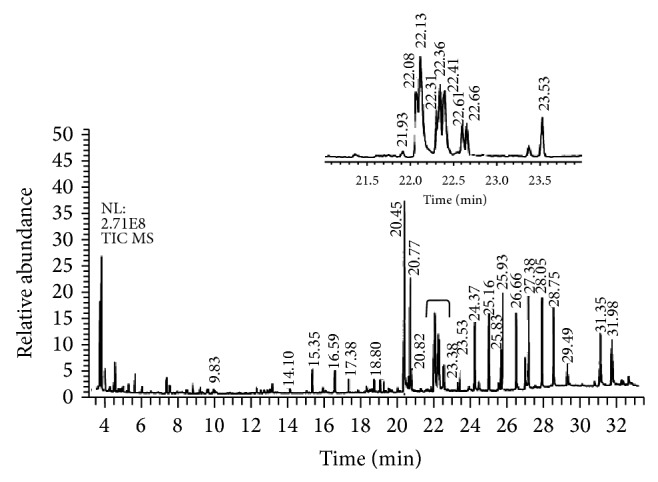
Total ion chromatogram of BPE-CO_2_A extract. Identified compounds are described in Table 1.

In the total ion chromatogram of BPE-CO_2_A (Figure 1) the presence of several compounds such as phytol (0.139%) and fatty acids such as palmitic (30%), oleic (27%), linoleic (24.3%), and linolenic (3.8%) acids was observed. In addition the following was detected in these plant extracts: thirteen alkanes (C_11_, C_14_, C_18_, C_20_, C_22_, C_23_, C_24_, C_25_, C_26_, C_27_, C_28_, C_29_, and C_30_), two ethyl esters of fatty acids (ethyl hexadecanoate and ethyl 9,12-octadecadienoate), two sterols (stigmasterol and sitosterol), and six unidentified compounds (Table 1).

### 3.2. BPE-CO_2_A Increases Extracellular Matrix Elements

**Figure 2:**
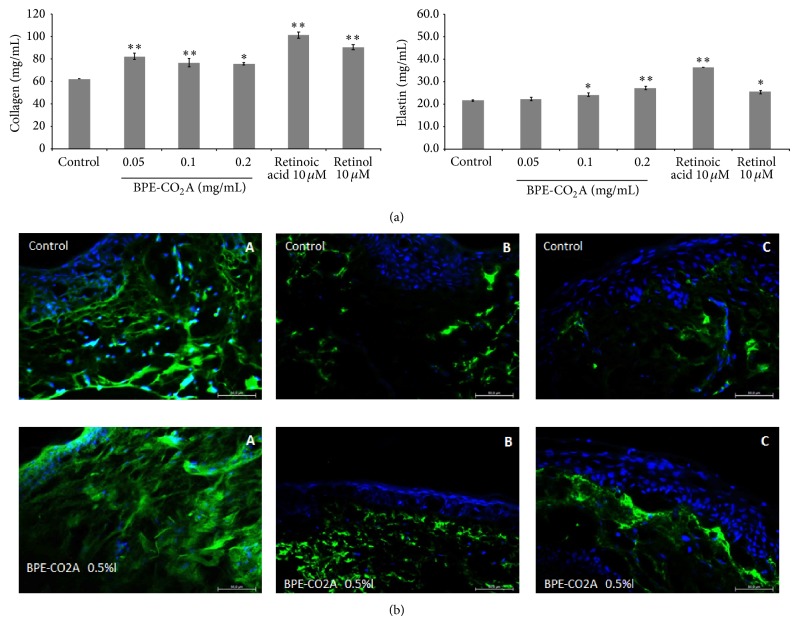
Extracellular matrix elements evaluation. (a) Collagen and elastin production in human fibroblast cultures after 48 hours of incubation with BPE-CO_2_A, retinoic acid, or retinol. (b) Collagen (A), elastin (B), and glycosaminoglycan (C) in human skin fragments labelled with anti-collagen (green), anti-elastin (green), and anti-glycosaminoglycan (green) antibodies, respectively, after a 48-hour incubation. The nuclei were counterstained with DAPI (blue) (40x magnification). The data represent the mean ± SD of three individual experiments. ^∗^ *P* < 0.05, ^∗∗^ *P* < 0.01.

To assess the retinoid-like activity of the* B. pilosa* extract, we analysed the synthesis of the collagen, elastin, and glycosaminoglycans in cultured human fibroblasts treated with various concentrations of* B. pilosa* extract, retinoic acid (10 *μ*M), or retinol (10 *μ*M). After treatment with BPE-CO_2_A, collagen levels were elevated by 32.5% at 0.05 mg/mL, 23.3% at 0.1 mg/mL, and 21.6% at 0.2 mg/mL. For comparison, retinoic acid (10 *μ*M) and retinol (10 *μ*M) were able to increase collagen levels by 62.9% and 45.6%, respectively (Figure 2). BPE-CO_2_A was also able to significantly increase the synthesis of elastin* in vitro*. At 0.2 mg/mL, the extract caused a 25.2% increase in elastin levels (Figure 2). As expected, retinoic acid (10 *μ*M) and retinol (10 *μ*M) increased elastin production by 66.7% and 16.7%, respectively. In contrast, BPE-CO_2_A was unable to significantly alter the levels of glycosaminoglycans (data not shown). Corroborating with the* in vitro* results, BPE-CO_2_A increased the immunofluorescent staining for collagen and elastin (Figures 2(b)(A) and 2(b)(B)). In addition, we also observed an increase in immunofluorescent staining for glycosaminoglycan when skin fragments were treated with BPE-CO_2_A (Figure 2(b)(C)).

### 3.3. BPE-CO_2_A Stimulates Growth Factors

**Figure 3:**
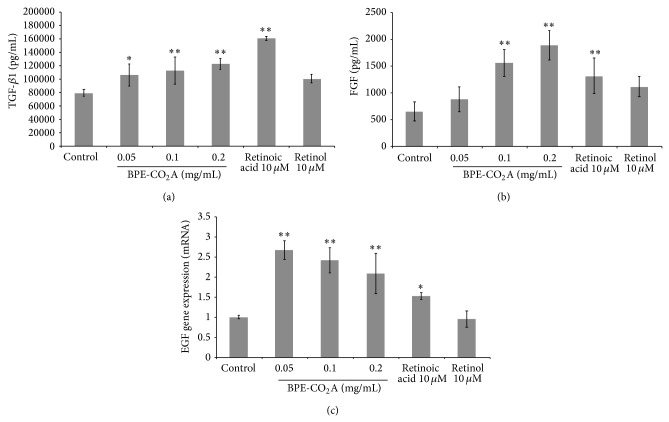
Effects of BPE-CO_2_A, retinoic acid, and retinol on the TGF-*β*1 (a) and FGF (b) levels and EGFr mRNA expression in human fibroblast cultures. The data represent the mean ± SD. ^∗^ *P* < 0.05, ^∗∗^ *P* < 0.01.

In another set of experiments, we evaluated the effects of BPE-CO_2_A on TGF-*β*1 and FGF levels in cultured human fibroblasts as well as the modulation of epidermal growth factor receptor (EGFr). At the three concentrations tested, BPE-CO_2_A significantly increased TGF-*β*1 levels, with its highest stimulatory effect (53.2%) at a concentration of 0.2 mg/mL (Figure 3). In the same assay, retinoic acid (10 *μ*M) increased TGF-*β*1 synthesis by 101.3%, and retinol was ineffective (Figure 3). Similar to its TGF-*β*1 induction, BPE-CO_2_A had a significant stimulatory effect on FGF levels (Figure 3) with an increase of 188.3% after the treatment of cultured human fibroblasts with BPE-CO_2_A at 0.2 mg/mL. Retinoic acid (10 *μ*M) and retinol (10 *μ*M) also caused significant FGF increases, but these effects were lower than those produced by BPE-CO_2_A (Figure 3). In addition, we evaluated the modulation of epidermal growth factor receptor (EGFr) gene expression. BPE-CO_2_A produced a 2.6-, 2.3-, and 2-fold increase in EGFr gene expression at concentration of 0.05 mg/mL, 0.10 mg/mL, and 0.2 mg/mL, respectively. Retinoic acid increased EGFr mRNA levels by 52.3%, and retinol was not able to significantly increase the expression of EGFr (Figure 3).

### 3.4. BPE-CO_2_A Modulates Retinoic Receptors

**Figure 4:**
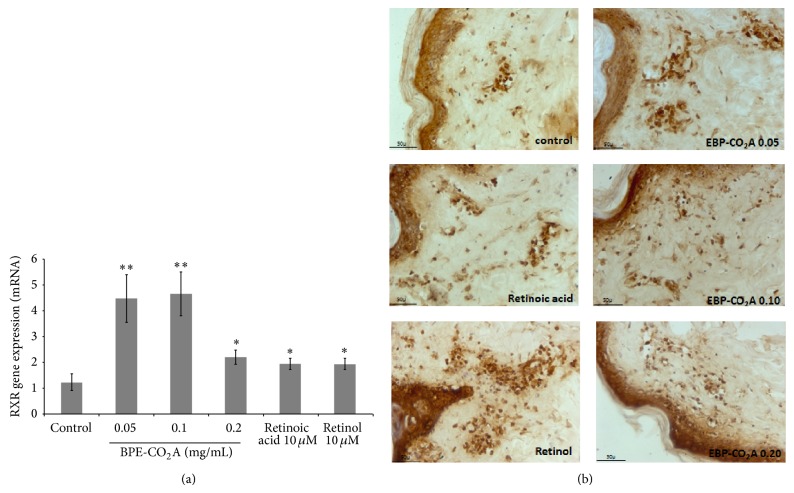
Effects of BPE-CO_2_A, retinoic acid, and retinol on RXR mRNA expression in human fibroblast cultures (a) and RXR synthesis (brown precipitate) in human skin fragments labelled with anti-RXR antibodies (b) after a 48-hour incubation (40x magnification). The data represent the mean ± SD. ^∗^ *P* < 0.05, ^∗∗^ *P* < 0.01.

**Figure 5:**
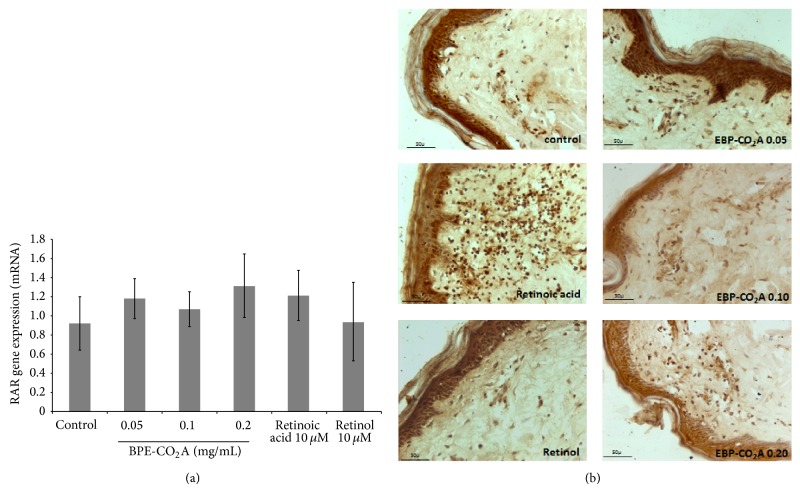
Effects of BPE-CO_2_A, retinoic acid, and retinol on RAR mRNA expression in human fibroblasts (a) and RAR synthesis (brown precipitate) in human skin fragments labelled with anti-RXR antibodies (b) after a 48-hour incubation (40x magnification). The data represent the mean ± SD. ^∗^ *P* < 0.05, ^∗∗^ *P* < 0.01.

To evaluate whether the effects induced by BPE-CO_2_A have similar effects to retinoic acid and retinol in the modulation of receptors RXR and RAR, we evaluated the ability of the extract to modulate the gene expression of these receptors in human fibroblasts (Figures 4 and 5). Gene expression of RXR increased 3.6-, 3.7-, and 1.6-fold in the group treated with BPE-CO_2_A at the concentration of 0.05, 0.10, and 0.20 mg/mL, respectively, whereas retinoic acid and retinol increased gene expression 1.5-fold (Figure 4(a)). An increase in receptor staining in the immunohistochemical evaluation of RXR performed on human skin fragments corroborates the* in vitro* results (Figure 4(b)).

In contrast, the expression of RAR was not significantly changed after treatment with BPE-CO_2_A, retinoic acid, or retinol (Figure 5(a)). However, when we evaluated RAR protein synthesis using immunohistochemistry, we found that treatment with retinoic acid markedly increased RAR staining, whereas treatment with BPE-CO_2_A and retinol promoted only subtle stimulation (Figure 5(b)).

**Figure 6:**
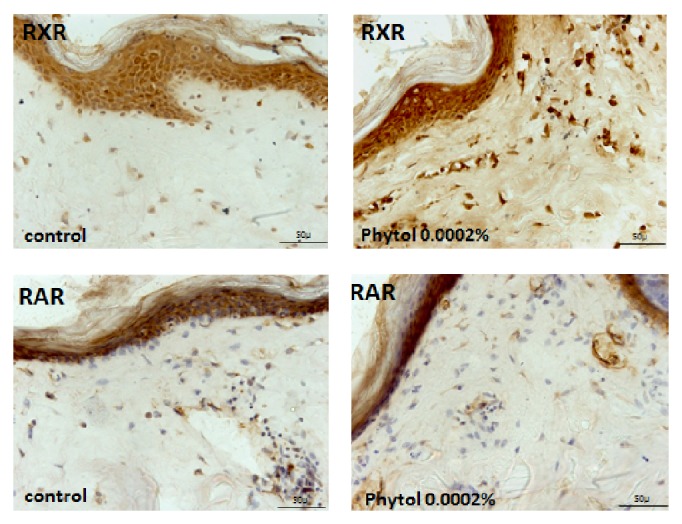
Effects of phytol on RXR and RAR synthesis (brown precipitate) in human skin fragments labelled with anti-RXR and anti-RAR antibodies after a 48-hour incubation (40x magnification).

We also evaluated the effect of phytol, an acyclic isoprenoid compound present in BPE-CO_2_A, on RAR and RXR production in skin fragments. Phytol (0.0002%) had similar effects to BPE-CO_2_A, inducing RXR synthesis (Figure 6) and not altering RAR production (Figure 6). It is thus possible that the effect of BPE-CO_2_A on retinoid receptors might be due to the presence of phytol.

### 3.5. BPE-CO_2_A Improves Histological Characteristics

**Figure 7:**
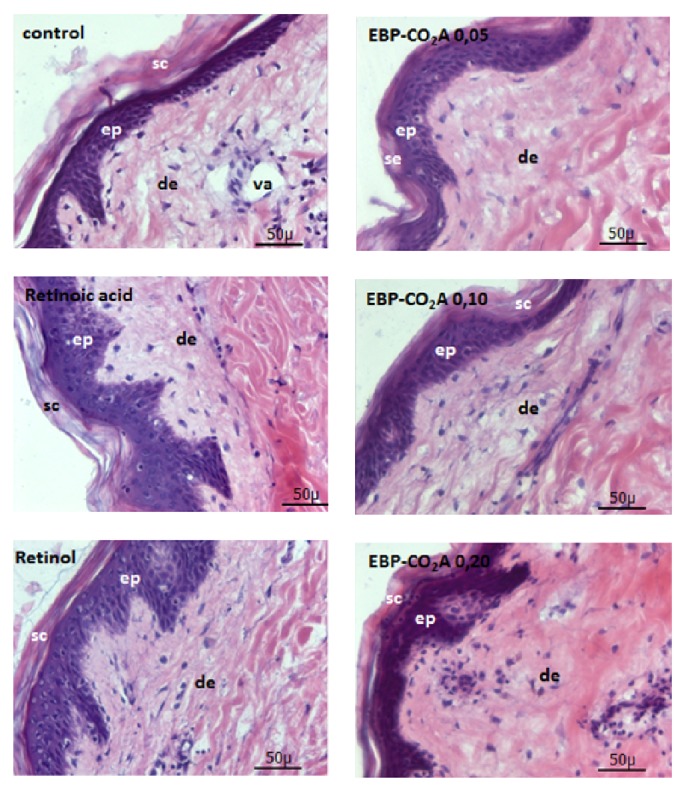
Photomicrographs of human skin fragments stained with haematoxylin-eosin and treated with retinoic acid, retinol, or BPE-CO_2_A (40x magnification). Sc: stratum corneum, ep: epidermis, de: dermis, and va: vacuoles.

Haematoxylin-eosin staining of human skin fragments was performed after various treatments with retinoic acid, retinol, and BPE-CO_2_A (Figure 7). A comparative analysis showed that the control group has a viable epidermal structure but a less cohesive and thinner stratum corneum than the treated groups. In this group, some vacuoles were observed, resulting in a less dense dermis. Retinoic acid exhibits its classical effects, such as the smooth peeling that improves the characteristics of the epidermal basal layer (viable cells), after a single application. The group treated with retinol had characteristics similar to those observed after treatment with retinoic acid, but with a smaller disruption of the horny layer, due to less aggressive behaviour towards retinoic acid and retinol. BPE-CO_2_A, in turn, had a slight effect in increasing the thickness of the epidermal basal layer and dermis (Figure 7).

## 4. Discussion 

The ageing of human skin is a complex biological phenomenon consisting of two components: intrinsic ageing (passage of time and individual genetic features) and extrinsic ageing caused by cumulative exposure to environmental factors such as ultraviolet radiation [21]. Intrinsic ageing is a slow, cumulative, progressive, and degradative process affecting mainly elastic fibre, while in extrinsic ageing the slow evolution can be enzymatically accelerated [22]. Both skin ageing processes are associated with structural and functional changes that occur in the dermal extracellular matrix, where fibrillar collagens, elastic fibres, and glycosaminoglycans are necessary to confer tensile strength, resilience, and hydration [23]. In both intrinsic and extrinsic ageing, there is evidence for the degradation of fibrous extracellular matrix components including elastin, collagens, and the oligosaccharide fraction [23, 24]. Our results showed that BPE-CO_2_A was able to significantly increase the synthesis of collagen and elastin* in vitro.* Although the* in vitro* stimulation promoted by BPE-CO_2_A on extracellular matrix components is quantitatively lower than the effect exerted by retinoic acid, it is evident in the* ex vivo* experiments that BPE-CO_2_A stimulates the production of collagen and elastin in the skin.

The fundamental mechanism for age-related collagen synthesis involves the transforming growth factor-*β* (TGF-*β*) signalling pathway, which stimulates collagen synthesis in cultured fibroblasts [25–28]. Moreover, it has been suggested that reactive oxygen species can activate different surface receptors, including those for epidermal growth factor, and this effect may be mediated in part by the mitogen-activated protein kinase (MAPK) pathway [22, 29]. The activation of several MAPK members, particularly c-Jun and c-Fos, is related to the transcription complex AP-1 that directly inhibits the production of procollagen by blocking the TGF-*β* receptor [30, 31]. Topical TGF-*β* treatment in wounds promotes collagen synthesis, increases tensile wound strength, stimulates granulation tissue formation, enhances the thickness of regenerate dermal tissue, and stabilizes the dermoepidermal junction [32, 33]. It has been related that the reduction of collagen in photoaged human skin occurs due to blocking the TGF-*β* receptor [30]. BPE-CO_2_A, retinoic acid, and retinol enhanced the production of TGF-*β* in fibroblast cultures, and this effect was in synergy with the reduction in collagen production that we detected in our experiments. Our results clearly showed that the increase in collagen synthesis is closely related to the enhanced production of TGF-*β*.

In addition to TGF-*β*, fibroblast growth factor-1 has a wide activity spectrum, mainly increasing synthesis of matrix macromolecules, notably the main dermal glycosaminoglycan, hyaluronic acid [34], which contributes to the wound-healing process by stimulating fibroblast proliferation and inhibits the expression of collagenase-1 in keratinocytes [35]. In our study, BPE-CO_2_A exerted a potent stimulatory effect on FGF-*β* levels in cultured human fibroblasts that was higher than the effects produced by both retinoic acid and retinol.

Another representative member of the growth factor family is epidermal growth factor (EGF), which can bind to and activate members of the EGF receptor (EGFR) family, leading to the initiation of several MAPKs signal transduction cascades and playing an important role in many cellular functions, such as proliferation, apoptosis, migration, inflammation, and immunity [36–39]. In fact, EGF induces collagenase expression in human dermal fibroblasts with a consequent reduction in collagen synthesis [40, 41]. Our results showed that BPE-CO_2_A was able to increase EGFR gene expression (2.6-fold), although retinoic acid produced a smaller increase (53.6%), and retinol was not able to increase gene expression. Thus, it is clear that the protective effect of BPE-CO_2_A on collagen and elastin degradation was closely related to the increase in the expression of growth factors, particularly TGF-*β* and FGF-*β*, and EGFR expression in human fibroblasts. This effect was similar to that produced by retinoic acid and was sometimes more marked than effects produced by both retinoic acid and retinol.

In a second set of experiments, we evaluated the effects of BPE-CO_2_A on both the gene expression and synthesis of the retinoic acid and retinoid X receptors. BPE-CO_2_A, retinoic acid, and retinol were not able to significantly alter the gene expression of RAR. However, retinoic acid markedly increased RAR protein synthesis as evidenced using immunohistochemistry, whereas BPE-CO_2_A treatment produces a subtle stimulation, similar to that produced by retinol. In contrast, BPE-CO_2_A treatment increased RXR gene expression significantly (3.6-fold compared to control) and to a greater extent than retinoic acid and retinol, both of which increased RXR gene expression approximately 1.5-fold. This effect was corroborated by an increase in RXR protein synthesis.

There is evidence that RAR and RXR mRNA levels decrease with age and that compounds able to increase the expression of these genes have neuroprotective effects in ageing [42, 43]. Thus, expression of RXRs was reduced in healthy elderly men suggesting that RXRs reduction might be a common feature of physiological senescence [44]. In addition, a single dose of all-*trans* retinoic acid to old rats was found to increase RAR expression in the liver after 24 h, indicating that reversible alterations in retinoid receptors may also occur during ageing [44, 45]. This way, it is possible that the protective effects produced by BPE-CO_2_A are also related to RXR mRNA expression.

Finally, BPE-CO_2_A contains a mixture of phytol and several long chain fatty acids, mainly palmitic, oleic, linoleic, and linolenic acids. Phytol, a branched fatty alcohol, is a carbon side-chain of chlorophylls and their metabolites, such as phytanic acid. Phytol has been shown to be a natural ligand for RXR, able to mimic various effects of conjugated linolenic acids, which activate the peroxisome proliferator-activated receptor (PPAR) and the RXRs [46, 47]. There is evidence that PPAR-*γ* mRNA levels decrease with age [41] and that phytol is functional as a PPAR-*α* ligand, stimulating the expression of PPAR-*α*-target genes in intact cells [47]. Moreover, phytol metabolites are compelling candidates for physiological effectors because their RXR binding affinities and activation potencies match their micromolar circulating concentrations [46, 48]. In contrast, reduced concentrations of long-chain fatty acids have been correlated with normal ageing and neurodegeneration [48], and some of these long-chain fatty acids are also RXR ligands or increase RXR gene expression [12–16, 49]. In our study, phytol was able to induce RXR synthesis but was not able to influence RAR production. Because phytol is exclusively of dietary origin, a* Bidens pilosa *extract containing phytol may represent an essential dietary product to control cellular metabolism through the RXR signalling pathways.

## 5. Conclusion

In conclusion, our results showed that BPE-CO_2_A from* Bidens pilosa* enhanced extracellular matrix components, particularly collagen and elastin fibres through the maintenance of TGF-*β*, FGF-*β*, and EGF levels and, simultaneously, affected RXR gene expression and synthesis. It is an innovative active ingredient with several clinical applications and pharmacological activities that control intrinsic and extrinsic human skin ageing. This way, BPE-CO_2_A from* Bidens pilosa* is an innovative active ingredient with potential clinical applications to control intrinsic and extrinsic human skin ageing and to develop an herbal preparation with retinoid-like activity useful as a skin-repair and antiageing agent.

## Figures and Tables

**Table 1:** Chemical compounds identified in the BPE-CO_2_ extract by gas chromatography and mass spectroscopy (GC/MS) according to chromatogram from Figure 1.

Retention time (min)	Compounds	Molecular formula
9.83	Undecane	C_11_
14.10	Tetradecane	C_14_
15.35	Unidentified	
16.59	Unidentified	
17.38	Unidentified	
18.80	Octadecane	C_18_
20.45	Hexadecanoic acid (palmitic acid)	C_16_H_32_O_2_
20.77	Ethyl hexadecanoate	C_18_H_36_O_2_
20.82	Eicosane	C_20_
21.93	3,7,11,15-Tetramethyl-2-hexadecen-1-ol (phytol)	C_20_H_40_O
22.08	9,12-Octadecadienoic acid (linoleic acid)	C_18_H_32_O_2_
22.13	9-Octadecenoic acid (oleic acid)	C_18_H_34_O_2_
22.31	Octadecanoic acid (stearic acid)	C_18_H_36_O_2_
22.36	Ethyl 9,12-octadecadienoate	C_20_H_36_O_2_
22.41	9,12,15-Octadecatrienoic acid (linolenic acid)	C_18_H_30_O_2_
22.61	Unidentified	
22.66	Docosane	C_22_
23.53	Tricosane	C_23_
24.37	Tetracosane	C_24_
25.16	Pentacosane	C_25_
25.83	Unidentified	
25.93	Hexacosane	C_26_
26.66	Heptacosane	C_27_
27.38	Octacosane	C_28_
28.05	Nonacosane	C_29_
28.75	Triacontane	C_30_
29.49	Unidentified	
31.35	Stigmasterol	C_29_H_48_O
31.98	Sitosterol	C_29_H_50_O
